# Innovations in Quality Improvement Research for more useful answers to research users’ questions

**DOI:** 10.1186/1748-5908-8-S1-S7

**Published:** 2013-04-19

**Authors:** Danielle Olds, John Øvretveit

**Affiliations:** 1 Veterans Affairs National Quality Scholars Program, Louis Stokes Cleveland VA Medical Center, Cleveland, Ohio, 44106, USA; 2 Medical Management Centre, The Karolinska Institutet, Stockholm, SE-171 77, Sweden

## Presentation

Quality Improvement Research (QIR) is any systematic inquiry that generates actionable knowledge, enables practitioners and patients to improve care and health, and reduces bias to maximize the validity and reliability of the knowledge gained. Some QIR evaluates complex healthcare innovations, such as bundled interventions to reduce infections. Such innovations often involve multiple components (e.g., hand hygiene, review of device necessity), may be directed at various levels of the system (e.g., practitioner, provider teams, hospital units), and may change in content or implementation strategy across time and context. Such innovations are affected by contextual factors emanating from different levels of the system.

If QIR addresses practitioners’ questions, it is more likely to be used. There are seven questions relevant to quality improvement (QI) practice: 1) Efficacy: does it work in controlled situations? 2) Effectiveness: will the intervention work in a setting and with patients like the practitioner’s own?; 3) Implementation: what are successful ways to implement change?; 4) Sustainability: what is needed to sustain the change?; 5) Fidelity: should the intervention be copied exactly?; which parts can be adapted?; 6) Cost: what are the costs and savings of making and sustaining the change?; and 7) De-implementation: how are ineffective practices modified?

Research designs and methods must be chosen to match research users’ needs. Designs frequently used in research, such as randomized controlled trials (RCTs), might not address questions of interest to stakeholders (e.g., clinicians, administrators, patients, payers). QIR can be broadened to include adherence studies and multi-morbidity studies, and to use budget impact analyses, action evaluation, research syntheses, and context-and-theory-informed program evaluations, as well as RCTs that incorporate process evaluations. Secondly, to be more relevant to current initiatives, such as Patient Centered Medical Homes, QIR could include estimates of costs and savings of improvements. Thirdly, QIR can be extended to non-hospital settings to study improvements that significantly affect patients’ health and cost. In these settings, QIR could be used to study epidemiology of adherence, conditions that facilitate or impede health behaviors, and interventions to improve self-care and self-management. Finally, an important point raised during conference discussion is the need to understand how practitioners and administrators use QIR. Because the goal of QIR is to provide actionable knowledge, researchers need to know how and even if, their findings are being used.

## Commentary

To advance QIR, there is a need to move beyond traditional QI methods (e.g., statistical process control charts) and traditional research methods (e.g., RCTs). Much QI research focuses on the question, “does it work?” by relying on RCTs and quasi-experimental methods, leaving other important questions uninvestigated. Meanwhile, QI practitioners often implement QI interventions with little evidence base. QIR could be advanced by being more closely tied to stakeholder questions and needs. If choice of QIR design and methods was driven by stakeholders’ questions, QIR may subsequently be more applied and innovative [1,2]. Adoption of methods used in other disciplines, such as implementation science and program evaluation, may close these shortcomings. Further, these methods may become even more important as QIR moves beyond the hospital and clinic and into the home and community settings [3].

Questions of costs and savings are important to stakeholders, but are often unaddressed in QIR. This is particularly relevant considering recent European austerity measures cost reduction measures in U.S. healthcare reform. There is a growing expectation in academic journals that QIR reports include at least a description of the resources required. In complex interventions, resource utilization can include costs and savings for the system overall and at different time-spans. These include not only the initial outlay for the intervention, but also the costs to sustain the intervention over time and their distribution across different stakeholders. Improvements in one part of the system may result in a volume decline and less reimbursable activity in another part of the system. Of the six abstract presentations for this conference, one mentioned the need to evaluate cost, but none presented an assessment of the costs and savings for stakeholders. Cost effectiveness analyses are beginning to be used, but there remains a lack of resource assessments and budget impact studies.

Because of the need and expectation of QIR to answer research users’ questions, QI researchers must partner with stakeholders to understand how QIR is used in practice. QI researchers could partner with frontline clinicians in the design, implementation, and evaluation of innovations. This could also occur through direct communication and dissemination through practice-based conferences and publications. Deeper knowledge about how QIR findings are used in practice, education, and policy could lead to study designs that answer research users’ question and findings that directly impact healthcare quality and cost.

## Recommendations

QI practitioners and managers are not waiting for research, and are often disappointed with the relevance of the research that is carried out. More innovative and relevant QIR is needed to provide an evidence base to usefully answer users’ questions so that their decisions are more effective. The first recommendation is to move QIR beyond its prior emphasis on efficacy to focus more attention on answering questions about effectiveness, implementation, sustainability, fidelity, de-implementation, and the costs and savings involved. The second recommendation is for QIR to explicitly include innovation costs and saving so as to meet the growing needs of healthcare systems to increase efficiency and contain expenditures. The third recommendation is that to be more useful, QIR needs to be applied to settings where the most healthcare occurs, which are in patients’ homes and communities. The last recommendation to make QIR more useful in addressing users’ questions is to understand how QIR is used in practice, education, and policy.
